# Improving the safety of outpatient parenteral antimicrobial therapy for patients with solid tumors

**DOI:** 10.1007/s00520-021-06549-3

**Published:** 2021-09-22

**Authors:** Alison Robins, Emma Dishner, Patrick McDaneld, Meagan Rowan, Jalen Bartek, Ying Jiang, Javier Adachi, Natalie J. M. Dailey Garnes

**Affiliations:** 1 Division of Internal Medicine, Department of Infectious Diseases, Infection Control and Employee Health, The University of Texas MD Anderson Cancer Center, Houston, TX USA; 2 Section of Infectious Diseases, Department of Medicine, Baylor College of Medicine, Houston, TX USA; 3 Present Address: Infectious Diseases, Medical Care Line, Michael E. Debakey VA Medical Center, Houston, TX USA; 4 Present Address: Division of Infectious Diseases, Baylor University Medical Center, Dallas, TX USA; 5 Division of Pharmacy, The University of Texas MD Anderson Cancer Center, Houston, TX USA

**Keywords:** Outpatient parenteral antimicrobial therapy, OPAT, Oncology, Hospital readmissions, Laboratory monitoring, Follow-up

## Abstract

**Background and objectives:**

Outpatient parenteral antimicrobial therapy (OPAT) for infections has been in use for nearly 40 years, and although it has been found safe and efficacious, its use has been studied primarily among otherwise healthy patients. We aimed to develop and evaluate an OPAT program for patients with cancer, particularly solid tumors.

**Methods:**

We implemented multiple quality improvement interventions between June 2018 and January 2020. We retrospectively and prospectively collected data on demographics, the completeness of infectious diseases (ID) physician consultation notes, rates of laboratory test result monitoring, ID clinic follow-up, and 30-day outcomes, including unplanned OPAT-related readmissions, OPAT-related emergency center visits, and deaths.

**Results:**

Completeness of ID provider notes improved from a baseline of 77 to 100% (*p* < .0001) for antimicrobial recommendations, 75 to 97% (*p* < .0001) for follow-up recommendations, and 19 to 98% (*p* < .0001) for laboratory test result monitoring recommendations. Completion of laboratory tests increased from a baseline rate of 24 to 56% (*p* = .027). Thirty-day unplanned OPAT-related readmission, ID clinic follow-up, 30-day emergency center visit, and death rates improved without reaching statistical significance.

**Conclusions:**

Sustained efforts, multiple interventions, and multidisciplinary engagement can improve laboratory test result monitoring among solid tumor patients discharged with OPAT. Although demonstrating a decrease in unplanned readmissions through institution of a formal OPAT program among patients with solid malignancies may be more difficult compared with the general population, the program may still result in improved safety.

## Introduction

Outpatient parenteral antimicrobial therapy (OPAT), first described in the 1970s, has increasingly been used as an alternative to hospitalization for stable patients with infections not amenable to oral therapy [1, 2]. OPAT has been associated with improved patient satisfaction, improved quality of life, reduced length of hospital stay, and successful treatment in 88–92% of cases [3–6]. Despite these benefits, up to 1 in 4 patients are readmitted within 30 days after discharge [7–11]. The Infectious Diseases Society of America initially published guidelines for OPAT in 2004 with updates in December 2018. These guidelines recommend consultation with a physician specializing in infectious diseases (ID), outpatient follow-up, and periodic monitoring of laboratory test results, with the goal of reducing adverse events [12, 13]. Since the publication of these guidelines, studies have reported reduced readmissions among patients who follow up with an ID specialist or in an OPAT clinic within 2–4 weeks of hospital discharge [9, 14]. Lack of laboratory test result monitoring has also been shown to be independently associated with hospital readmission [8].

Although studies and guidelines addressing the safe delivery of OPAT exist, a knowledge gap remains regarding the effectiveness of OPAT in certain patient populations. For example, while studies investigating OPAT among persons who inject drugs have shown outcomes, including response to therapy and catheter-related adverse events, are similar to patients without a history of intravenous drug use, additional special populations exist that require further study [15]. For example, the effectiveness of OPAT among patients with oncologic diagnoses and receiving antineoplastic therapy requires further exploration. Despite recent developments, cancer therapeutics, or their sequelae, continue to confer varying degrees of immunosuppression, resulting in an increased risk for infection [16–19]. Most studies specifically focused on patients with oncologic diagnoses receiving OPAT have focused on the treatment of febrile neutropenia [20–25]. The current report discusses our quality improvement experience in patients with solid tumors receiving OPAT at a single comprehensive cancer center.

## Methods

### Interventions

We retrospectively and prospectively reviewed charts of patients with solid tumors at our institution who were discharged with OPAT at the recommendation of an inpatient ID consultation between November 13, 2017, and January 5, 2020. When patients were readmitted while receiving OPAT and the OPAT was continued, we considered this a single OPAT episode. All patients had been admitted to a solid tumor medical or surgical oncology service. We focused on patients with solid tumors as a pilot program with the goal to expand OPAT to additional patient populations. Patients admitted to services focusing on leukemia, lymphoma, myeloma, pediatrics, stem cell transplantation, and oncology hospitalist services were excluded from the current pilot. We also excluded patients discharged to hospice from their otherwise qualifying admission and patients who completed the recommended antimicrobial regimen while admitted or transitioned to oral therapy upon discharge. We included patients discharged to other facilities. At our institution, primary teams write outpatient antimicrobial prescription orders, including for our institutional infusion center, and case management teams arrange care with external outpatient care providers (e.g., a home health agency, home infusion company, or outpatient infusion center).

This quality improvement project was approved by our institutional Quality Improvement Assessment Board and deemed not to be research (QIAB #269). We collected pre-intervention data retrospectively by identifying patients who met our inclusion criteria and had an ID consultation between November 13, 2017, and January 8, 2018 (i.e., the pre-intervention phase). During our intervention phases, which in total spanned from June 4, 2018, through January 5, 2020, we collected patient information prospectively and retrospectively. Our manuscript was prepared in consideration of SQUIRE 2.0 guidelines [26].

**Fig. 1:**
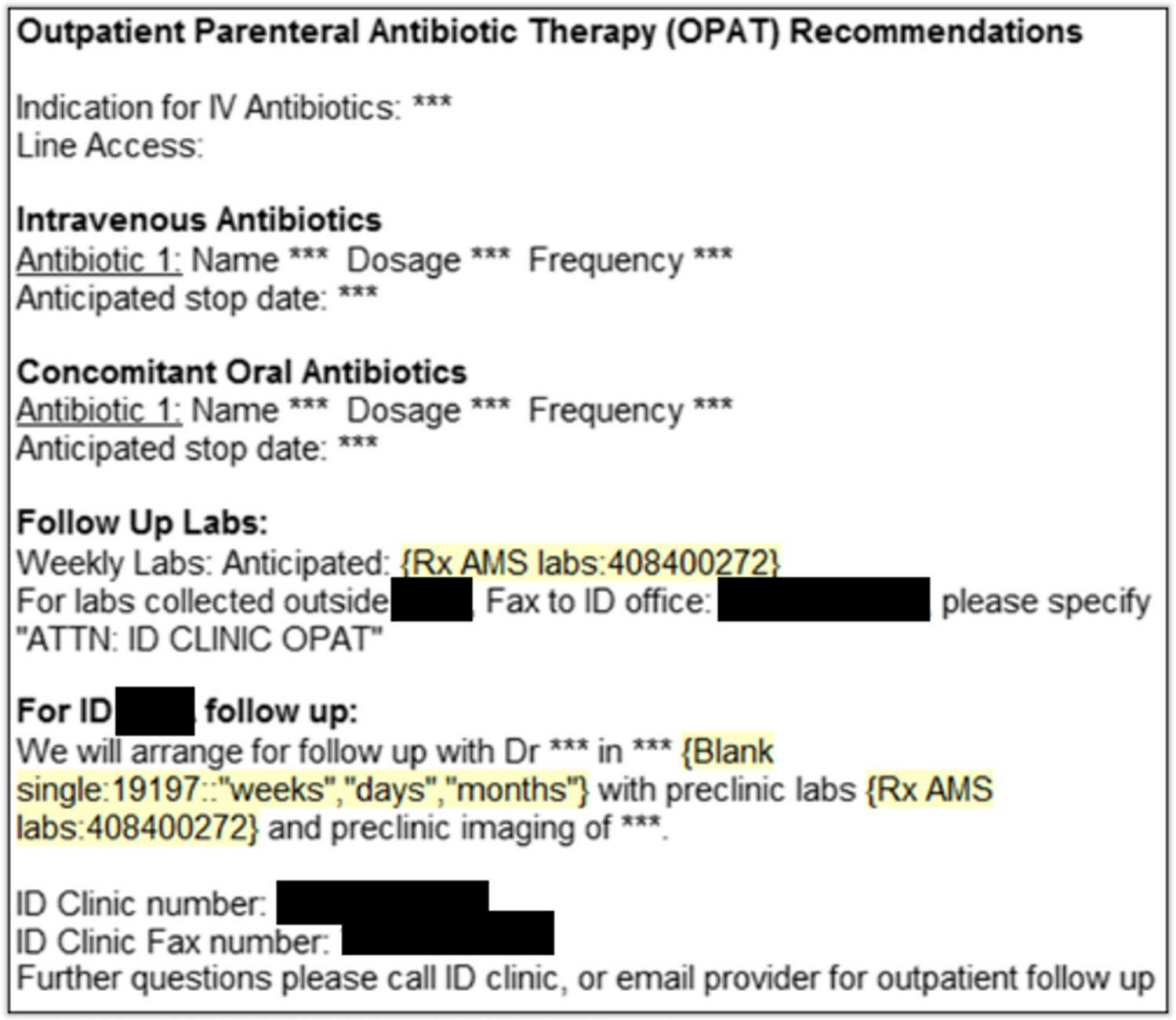
Standardized electronic medical record template for infectious diseases provider recommendations, introduced as part of our phase 1 intervention and used in subsequent interventions

The Plan-Do-Study-Act methodology guided patient safety improvements. By brainstorming and constructing fishbone diagrams, we identified obstacles to patient follow-up in the ID clinic and monitoring laboratory test results. We mapped the OPAT referral process to identify and prioritize opportunities for improvement. We found that many obstacles to patient follow-up could be attributed to unclear ID consultation recommendations. Thus, we initially sought to clarify recommendations by creating a standardized template within our electronic medical record (EMR) software to be used when making these recommendations to ensure their completeness, including antimicrobial name, dose, duration, frequency, follow-up time frame, and monitoring labs (Fig. 1). ID providers received weekly reminders regarding the new process during the phase 1 intervention period, which was June 4, 2018, through July 15, 2018. Furthermore, ID providers were asked to securely email our team and outpatient clinical nursing staff the Medical record numbers (MRNs) of patients for whom OPAT was recommended to facilitate prospective data collection and transition of care. Concomitantly, outpatient nurses tracked patients for timely follow-up and review of laboratory test results. During the post-phase 1 intervention period, which spanned from July 16, 2018, through January 31, 2019, ID providers received monthly, rather than weekly, reminders to follow these procedures.

Our subsequent intervention phases continued the phase 1 intervention while adding new interventions. Our phase 2 intervention, which spanned from February 1, 2019, through May 14, 2019, focused on engaging the case management teams, so that the process map now also included external home health and infusion agencies and laboratories. For this intervention, we asked the primary inpatient team to copy and paste the standardized template into a case management consultation order. For our phase 3 intervention, which spanned May 15, 2019, through January 5, 2020, we asked ID providers to order the recommended recurring laboratory tests to be monitored for the duration of therapy. For both phase 2 and phase 3 interventions, ID providers received monthly reminders to follow these procedures.

In addition to these phased interventions for ID providers, we further involved Advanced practice providers (APPs) (i.e., advanced practice nurses and physician assistants practicing ID) in the transition from inpatient to outpatient care. We developed a new APP OPAT clinic, where patients could be seen earlier than their scheduled follow-up appointment with an ID physician. APPs also called patients to query and document side effects and central line complications, as well as to confirm that recommended laboratory tests were being performed. These telephone visits were conducted between hospital discharge and ID clinic follow-up while patients were receiving OPAT.

### Data collection

The inpatient ID consultation team securely emailed MRNs of patients for whom OPAT was recommended to the OPAT team. Data was collected and managed using REDCap (Research Electronic Database Capture) secure, web-based electronic software data capture tools hosted at The University of Texas MD Anderson Cancer Center [27, 28]. Coinvestigators reviewed patient data to document admission and discharge dates; date of final ID consultation recommendations; age; sex; type of malignancy; indication for OPAT; patient disposition, including location of OPAT administration; outcomes. Eastern Cooperative Oncology Group performance status (ECOG-PS), a measure of patient functional status [29]; disease stage; Charlson comorbidity index [30]; vascular access; insurance status; Multi-drug resistant organism (MDRO), Vancomycin-resistant enterococcus (VRE), and Methicillin-resistant *Staphylococcus aureus* (MRSA) infection; and the presence of febrile neutropenia during the qualifying admission were obtained by electronic query of the EMR. Vascular access was classified as Peripherally inserted central catheter (PICC), subcutaneous port, or other, which included tunneled central venous catheter, non-tunneled central venous catheter, or peripheral IV only. MDRO includes any Gram negative bacillus, except *Stenotrophomonas maltophilia,* resistant to 3 of the 4 among ceftazidime or cefepime, imipenem or meropenem, piperacillin/tazobactam, ciprofloxacin or levofloxacin; *Stenotrophomonas maltophilia* resistant to trimethoprim/sulfamethoxazole; and *Streptococcus pneumoniae* resistant to 2 of the 3 among penicillin, parenteral, non- cerebrospinal fluid (CSF), ceftriaxone, non-CSF, fluoroquinolones. Additionally, we started collecting data on patient-reported adverse outcomes and antimicrobial classes received, as documented in the EMR, during phase 2 and phase 3 interventions, and after the phase 3 intervention; therefore, this data was available only for a subset of patients.

We monitored the completeness of ID consultation recommendations, focusing on the recommended antimicrobial regimen, follow-up schedule, and laboratory studies. Primary outcomes included completion of laboratory tests and ID clinic follow-up as recommended by the ID physician, measured as percentages of patients completing laboratory tests and follow-up. Laboratory tests were considered complete if available for review in the EMR and could have been completed specifically for OPAT or as part of patient’s primary oncology follow up. Secondary outcomes included documented completion of antimicrobial regimens, 30-day unplanned OPAT-related readmissions, OPAT-related Emergency center (EC) visits, and deaths.

To measure primary and secondary outcomes, we reviewed patient charts for at least 30 days after discharge and through completion of OPAT if longer than 30 days. Antimicrobial regimen completion, readmissions, EC visits, deaths, and duration of OPAT were determined by documentation in the EMR. Patients who were readmitted during this 30-day period and then discharged to hospice were excluded from the 30-day outcome measure of death, because time of death was not always available in the EMR. Median duration of OPAT was calculated from the time of hospital discharge to completion of OPAT regimen and excluded days when patients were readmitted.

We also evaluated the impact of the program on costs, specifically hospital and provider charges, for 90 days after initial discharge with OPAT.

From January 2019 through June 2020 (i.e., during part of the post-phase 1 intervention, the phase 2 and phase 3 interventions, and after the phase 3 intervention), we administered surveys to a convenience sample of patients discharged with OPAT who returned to the ID clinic for follow-up. The surveys assessed patient opinions regarding communication effectiveness, hospital visits related to side effects, where they received antimicrobials and whether cost was a factor in that decision, time spent administering antimicrobials, and location of laboratory test administration.

### Statistical analysis

Categorical variables were compared using the chi-square or Fisher exact test, as appropriate. Continuous variables were compared using the Kruskal–Wallis test (for 5-group comparisons) and the Wilcoxon rank-sum test (for 2-group comparisons). If a significant result (*p* < 0.05) was detected for a test that compared five groups, pairwise comparisons were performed for each of the four intervention phases with the pre-intervention phase, with α levels adjusted using the Holm sequential Bonferroni adjustment to control type I error. Lastly, logistic regression analysis was used to identify the independent factors associated with unplanned OPAT-related 30-day readmission. Variables with *p*-values ≤ 0.25 on univariable analysis were considered in multivariable analysis. All tests were 2-sided with a significance level of 0.05, except the pairwise comparisons with the α adjustment. Statistical analyses were performed using SAS version 9.3 (SAS Institute Inc, Cary, NC).

## Results

### Patient characteristics

Patient characteristics are listed in Table 1 for each intervention phase. The median age of all patients was 61 years, without any significant difference among intervention phases. The distribution of types of malignancy was mostly similar among phases. The most common underlying malignancies included genitourinary (25.7%), breast (11.7%), gynecologic (11.7%), head and neck (11.2%), and sarcoma (10.7%). The ECOG-PS for most patients during each phase was 1–3, indicating slight restriction in activity to significant limitations in self-care. This remained stable across phases except for when comparing the pre-intervention phase to phase 2 (overall *p* = 0.045 with sub-comparison *p* = 0.002). PICC was the most used form of vascular access. Although a significant difference in other vascular access category was noted (*p* = 0.014), no significant difference was noted when comparing the pre-intervention phase to any other phase after correcting for multiple comparisons. The percentage of MDRO infections was 11–17%, and 5–13% of patients experienced MRSA infections without significant differences over the course of the project. Only four patients in the entire cohort experienced VRE infection. The percentage of patients experiencing febrile neutropenia during the qualifying admission ranged from 0 to 11%. The most common types of infections treated with OPAT included abscess (32.7%), bacteremia (28.9%), skin/soft tissue infections (25.9%), intra-abdominal infections (18.7%), and genitourinary infections (15.7%). OPAT indications were not mutually exclusive, and often intra-abdominal infections and skin/soft tissue infections included abscesses. Most patients received OPAT at home either with (50.4%) or without (29.7%) home health agency assistance as opposed to in an infusion center (9%) or long-term acute care facility (4.2%). Use of home health agency assistance increased over time during the intervention phases, although this trend did not reach statistical significance.

**Table 1:** Patient demographic information for each intervention phase^a^

Characteristic	No. (%)					*p-*value
	Pre-intervention phase, *n* = 48	Phase 1, *n* = 17	Post-phase 1, *n* = 149	Phase 2, *n* = 71	Phase 3, *n* = 116	
Median age (years) (range)	63 (27–87)	56 (39–76)	62 (21–84)	57 (20–83)	60 (21–90)	.75
Male	28 (58)	6 (35)	73 (49)	38 (54)	63 (54)	.47
Type of malignancy						
Anal	0 (0)	1 (6)	0 (0)	0 (0)	0 (0)	.042
Breast	3 (6)	3 (18)	21 (14)	9 (13)	11 (9)	.49
CNS^b^	3 (6)	2 (12)	14 (9)	5 (7)	9 (8)	.92
Gastrointestinal	3 (6)	2 (12)	26 (17)	4 (6)	12 (10)	.064
Genitourinary	16 (33)	7 (41)	32 (21)	18 (25)	30 (26)	.29
Gynecologic	9 (19)	0 (0)	20 (13)	8 (11)	10 (9)	.19
Head and neck	4 (8)	0 (0)	18 (12)	9 (13)	14 (12)	.58
Lung	3 (6)	1 (6)	5 (3)	1 (1)	2 (2)	.34
Sarcoma	4 (8)	1 (6)	8 (5)	10 (14)	20 (17)	.025
Skin	1 (2)	0 (0)	4 (3)	4 (6)	4 (3)	.79
Other	2 (4)	0 (0)	5 (3)	4 (6)	5 (4)	.91
Cancer stage						
Stage I	2/12 (17)	0/4 (0)	3/47 (6)	1/23 (4)	7/33 (21)	.19
Stage II	2/12 (17)	0/4 (0)	4/47 (9)	7/23 (30)	6/33 (18)	.18
Stage III	4/12 (33)	3/4 (75)	12/47 (26)	6/23 (26)	8/33 (24)	.32
Stage IV	4/12 (33)	1/4 (25)	28/47 (60)	9/23 (39)	12/33 (36)	.16
Insurance						.33
Commercial/Managed care	26 (54)	10 (59)	73/147 (50)	34 (48)	71/115 (62)	
Medicaid/Medicaid	2 (4)	0 (0)	4/147 (3)	1 (1)	1/115 (1)	
Managed care						
Medicare/Medicare	18 (38)	7 (41)	64/147 (44)	30 (42)	33/115 (29)	
Managed care						
Other	2 (4)	0 (0)	6/147 (4)	6 (8)	10/115 (9)	
Febrile neutropenia	1 (2)	0 (0)	6/147 (4)	8 (11)	11/115 (10)	.09
Charlson comorbidity index, median (range)	53 (0–98)	84 (0–98)	77 (0–98)	77 (0–98)	77 (0–98)	.96
ECOG-PS						.045
0	1/21 (5)	1/7 (14)	22/85 (26)	17/57 (30)	22/87 (25)	
1 2 3 4	4/21 (19) 7/21 (33) 9/21 (43) 0/21 (0)	1/7 (14) 1/7 (14) 3/7 (43) 1/7 (14)	23/85 (27) 22/85 (26) 17/85 (20) 1/85 (1)	23/57 (40) 7/57 (12) 9/57 (16) 1/57 (2)	29/87 (33) 19/87 (22) 15/87 (17) 2/87 (2)	
Vascular access^c^						
PICC	41 (85)	14 (82)	109/147 (74)	51 (72)	87/115 (76)	.45
Port	9 (19)	3 (18)	31/147 (21)	12 (17)	28/115 (24)	.78
Other	1 (2)	0 (0)	16/147 (11)	11 (15)	5/115 (4)	.014
MDRO	6 (13)	2 (12)	20/147 (14)	8 (11)	20/115 (17)	.79
MRSA	6 (13)	1 (6)	9/147 (6)	4 (6)	6/115 (5)	.51
OPAT indication						
Abscess	15 (31)	4 (24)	46 (31)	23 (32)	43 (37)	.75
Bacteremia	15 (31)	3 (18)	47 (32)	17 (24)	34 (29)	.64
CNS infection	5 (10)	4 (24)	18 (12)	9 (13)	10 (9)	.47
Endovascular infection	1 (2)	3 (18)	4 (3)	1 (1)	2 (2)	.041
Genitourinary infection	7 (15)	4 (24)	22 (15)	12 (17)	18 (16)	.91
Intra-abdominal infection	7 (15)	3 (18)	31 (21)	12 (17)	22 (19)	.89
Bone/joint infection	5 (10)	1 (6)	16 (11)	9 (13)	11 (9)	.93
Pneumonia	4 (8)	1 (6)	7 (5)	1 (1)	6 (5)	.52
Skin/soft tissue infection	12 (25)	2 (12)	38 (26)	20 (28)	32 (28)	.71
Other	1 (2)	0 (0)	4 (3)	2 (3)	7 (6)	.64
Disposition						
Home without home health agency assistance	19 (40)	6 (35)	51 (34)	18 (25)	25 (22)	.081
Home with home health agency assistance	21 (44)	7 (41)	70 (47)	34 (48)	70 (60)	.14
Nursing facility	2 (4)	2 (12)	11 (7)	5 (7)	7 (6)	.81
Long-term acute care	3 (6)	1 (6)	2 (1)	2 (3)	9 (8)	.06
Outpatient transfusion center	3 (6)	1 (6)	15 (10)	12 (17)	5 (4)	.052

^a^Pre-intervention phase: November 17, 2017–January 8, 2018; phase 1: June 4, 2018–July 15, 2018; post-phase 1: July 16, 2018–January 31, 2019; phase 2: February 1, 2019–May 14, 2019; phase 3: May 15, 2019–January 5, 2020

^b^Abbreviations:* CNS* central nervous system; *ECOG-PS* Eastern Cooperative Oncology Group performance status; *MDRO* multidrug resistant organism; *MRSA* methicillin-resistant *Staphylococcus aureus*; *OPAT* outpatient parenteral antimicrobial therapy; *PICC* peripherally inserted central catheter

^c^Vascular access may not sum to 100% as some patients had both PICC and port in place

### Completeness of notes

Regarding completeness of notes, complete antimicrobial recommendations were given for 37 of 48 pre-intervention phase patients (77%) compared with all 17 patients (100%) during the phase 1 intervention (Table 2). Rates of complete recommendations ranged from 94 to 100% during subsequent intervention phases. Regarding follow-up, complete recommendations were given, or follow-up was specifically not recommended, for 36 of 48 patients (75%) during the pre-intervention phase and for all 17 patients (100%) during the phase 1 intervention. The rates of complete follow-up recommendations varied from 83 to 99% during subsequent intervention phases. The proportion of patients for whom laboratory test recommendations were given, either recommendations for specific laboratory tests or a statement that laboratory tests were not needed, increased from a low of 9 of 48 patients (19%) during the pre-intervention phase to a maximum of 114 of 116 patients (98%) during the phase 3 intervention. Changes in all note completeness measures were statistically significant (*p* < 0.0001).

**Table 2:** Primary outcomes during each of the intervention phases^a^

Outcome	No. (%)					*p*
	Pre-intervention	Phase 1	Post-phase 1	Phase 2	Phase 3	
Completeness of notes						
No. of patients	48	17	149	71	116	
Complete antimicrobial recommendations^b^						
Yes	37 (77)	17 (100)	140 (94)	71 (100)	116 (100)	< .0001
Partial/no	11 (23)	0 (0)	9 (6)	0 (0)	0 (0)	
Recommended follow-up^c^						
Yes/not recommended	36 (75)	17 (100)	124 (83)	70 (99)	113 (97)	< .0001
Not discussed	12 (25)	0 (0)	25 (17)	1 (1)	3 (3)	
Recommended laboratory tests^d^						
Yes/not recommended	9 (19)	15 (88)	109 (73)	69 (97)	114 (98)	< .0001
Partial/no	39 (81)	2 (12)	40 (27)	2 (3)	2 (2)	
Primary outcomes						
Follow-up in infectious diseases clinic (if recommended)^e^						
No. of patients	31	15	103	55	85	
Yes	17 (55)	11 (73)	67 (65)	42 (76)	61 (72)	.25
Partial/no	14 (45)	4 (27)	36 (35)	13 (24)	24 (28)	
Completion of laboratory tests (if recommended)^f^						
No. of patients	21	15	111	68	113	
Yes	5 (24)	5 (33)	63 (57)	32 (47)	63 (56)	.027
Partial/no	16 (76)	10 (67)	48 (43)	36 (53)	50 (44)	

^a^Pre-intervention phase: November 17, 2017–January 8, 2018; phase 1: June 4, 2018–July 15, 2018; post-phase 1: July 16, 2018–January 31, 2019; phase 2: February 1, 2019–May 14, 2019; phase 3: May 15, 2019–January 5, 2020

^b^Yes indicates drug, dose, frequency, and duration were all present; partial indicates 3 of 4 components were present; no indicates two or more components were missing

^c^Includes provider and timeframe of follow-up

^d^Yes indicates laboratory test type, frequency, and contact information for results were all present; partial indicates 2 of 3 components were present; no indicates two or more components were missing

^e^Yes indicates that patient follow-up was completed within the recommended period; partial indicates that the patient followed up but later than recommended (8–30 days); no indicates that the patient did not follow up within 30 days of the recommended date. The analysis excluded patients for whom the infectious diseases physician either did not provide a recommendation about follow-up or specifically stated that no follow-up was needed. Follow-up by outside infectious diseases providers was considered complete only if we had documentation of this follow-up

^f^Yes indicates that the recommended laboratory tests at the recommended frequency were completed and results provided; partial indicates that some of the recommended laboratory tests were completed and results provided; no indicates that no laboratory tests were completed or results provided. The analysis excluded patients for whom the infectious diseases physician either did not provide recommendations about laboratory tests or specifically stated that no laboratory tests were needed

### Primary outcomes

The pre-intervention phase ID clinic follow-up rate was 55% (17 of 31 patients), and this increased to 73% (11/15 patients) during the phase 1 intervention and ranged from 65 to 76% during subsequent intervention phases, with no significant change over the project period (*p* = 0.25; Table 2). During the pre-intervention phase, completion of all recommended laboratory tests occurred in 5 of 21 patients (24%) and reached a maximum of 63 of 111 patients (57%) following the post-phase 1 intervention, with a significantly increased completion rate during the post-phase 1 intervention period (*p* = 0.006) and phase 3 intervention (*p* = 0.007).

### Secondary outcomes

We noted improvement in rates of documented completion of the recommended antimicrobial regimen from 65% (31 of 48 patients) in the pre-intervention phase to a maximum of 87% (62 of 71 patients) during the phase 2 intervention (Table 3). We found that 10 of 48 patients (21%) experienced unplanned OPAT-related readmissions within 30 days of discharge during the pre-intervention phase, 0/17 (0%) during the phase 1 intervention, with a low of 12/116 (10%) during the phase 3 intervention for the subsequent phases (*p* = 0.094). Univariable analysis identified ECOG-PS (*p* = 0.006), other vascular access (*p* = 0.042), gynecologic cancer (*p* = 0.024), incomplete antimicrobial recommendations (*p* = 0.044), and interventions prior to phase 3 (*p* = 0.037; ID providers began ordering monitoring labs during phase 3) as risk factors for readmission within 30 days of discharge. In multivariable analysis performed among all patients excluding ECOG-PS because of missing data, gynecologic cancer remained an independent risk factor for readmission [odds ratio (OR) 2.27, 95% confidence interval (CI) 1.10–4.68]. After adjusting for gynecologic cancer, intervention phase 3 continued to show a trend toward protection against readmission (OR 0.49, 95% CI 0.24–1.003). In a multivariable sub-analysis among patients with ECOG-PS available (*n* = 253), lung cancer (OR 27.46, 95% CI 1.27–593.51), intraabdominal infection (OR 3.71, 95% CI 1.60–8.61), dichotomous ECOG-PS 2–4 compared with 0–1 (OR 3.95 95% CI 1.70–9.17), other vascular access compared with PICC and/or port (OR 3.52, 95% CI 1.14–10.82), and phase 3 intervention compared with prior phases (OR 0.22, 95% CI 0.07–0.66) were independently associated with readmission. During the pre-intervention phase, 8 of 48 patients (17%) experienced OPAT-related EC visits within 30 days of discharge, and OPAT-related EC visits occurred for 0–21% of patients during the subsequent intervention phases (*p* = 0.26). We observed few deaths during the project period, with no statistically significant differences among the intervention phases (*p* = 0.46).

**Table 3:** Secondary outcomes during each of the intervention phases^a^

Outcome	No. (%)					*p*
	Pre-intervention, *n* = 48	Phase 1, *n* = 17	Post-phase 1, *n* = 149	Phase 2, *n* = 71	Phase 3, *n* = 116	
Confirmed completion of antimicrobials^b^						.018
Yes	31 (65)	13 (76)	119 (80)	62 (87)	99 (85)	
Partial/no/unknown	17 (35)	4 (24)	30 (20)	9 (13)	17 (15)	
30-day outcomes^c^						
Unplanned OPAT-related 30-day readmission/unknown	10 (21)	0 (0)	26 (17)	14 (20)	12 (10)	.094
Death	2/47 (4)	0 (0)	5/147 (3)	0/68 (0)	2/114 (2)	.46
OPAT-related EC visits/unknown	8 (17)	0 (0)	26 (17)	15 (21)	16 (14)	.26

^a^Pre-intervention phase: November 17, 2017–January 8, 2018; phase 1: June 4, 2018–July 15, 2018; post-phase 1: July 16, 2018–January 31, 2019; phase 2: February 1, 2019–May 14, 2019; phase 3: May 15, 2019–January 5, 2020

^b^Yes indicates that the patient received the correct antimicrobial(s) at the correct dose, frequency, and duration as recommended; partial indicates that the patient received the correct antimicrobial(s) but wrong dose, frequency, or duration; no indicates that the patient received the wrong antimicrobial(s) and/or 2 of the following were wrong: dose, frequency, or duration

^c^Abbreviations: *OPAT*, outpatient parenteral antimicrobial therapy; *EC*, emergency center. Patients enrolled in hospice were excluded from analysis of death as an outcome. For two patients each during the pre-intervention phase and phase 3 intervention, the reason for the EC visit and readmission was unknown

Median OPAT duration ranged from 10 to 13 days throughout the phase 1 through phase 3 interventions. Data on adverse events while receiving OPAT and the antimicrobials received were captured for 134 patients (Tables 4 and 5). Forty-six patients (34%) received two or more intravenous antimicrobials, and 71 (53%) received concurrent oral therapy. The most common antimicrobial classes prescribed were daptomycin (37%), carbapenems (37%), and cephalosporins (32%). We also evaluated control charts of costs for the 90 days following the index discharge with OPAT. Although we noted a decrease during the phase 1 intervention compared with the pre-intervention phase, this finding was not statistically significant and was not replicated during the phase 3 intervention or after the phase 3 intervention (data not shown).

**Table 4:** Adverse events reported during outpatient parenteral antimicrobial therapy, *n* = 134

Adverse event	No. (%)
Central line-associated bloodstream infection	4 (3)
Line-associated deep venous thrombosis	3 (2)
Allergic reaction	3 (2)
Nausea	15 (11)
Diarrhea	13 (10)
*Clostridioides difficile* infection	1 (1)
Laboratory test result abnormalities^a^	10 (7)
Other^b^	13 (10)

^a^Laboratory test result abnormalities included eosinophilia (2), hyponatremia (1), elevated alkaline phosphatase (1), hypokalemia (1), leukopenia (2; attributed to concomitant oral therapy in one case), and elevated creatine kinase (4)

^b^Other adverse events included fever/rigors (1), clogged line requiring emergency center visit (2), irritation at peripherally inserted central catheter line site without infection (1), slow flow through peripherally inserted central catheter line with inability to draw blood for laboratory tests (1), myalgias without elevated creatine kinase (1), headache (1), other central nervous system toxic effect (2), urticaria (1; antimicrobial regimen was not changed), neuropathy (1), edema (1), abnormal taste (1), and tinnitus/hearing loss (1)

**Table 5:** Intravenous antimicrobials received, *n* = 134

Antimicrobial class	No. (%)
Carbapenem^a^	49 (37)
Penicillin^b^	9 (7)
Cephalosporin^c^	43 (32)
Vancomycin	9 (7)
Daptomycin	49 (37)
New β-lactam/β-lactamase inhibitor^d^	5 (4)
Echinocandin^e^	16 (12)
Other^f^	15 (11)

^a^39 Ertapenem, 10 meropenem

^b^5 Ampicillin, 1 piperacillin/tazobactam, 1 ampicillin/sulbactam, 1 nafcillin, 1 oxacillin

^c^21 Ceftriaxone, 11 cefepime, 4 ceftazidime, 7 cefazolin

^d^2 Ceftazidime/avibactam, 2 ceftolozane/tazobactam, 1 meropenem/vaborbactam

^e^15 Caspofungin, 1 anidulafungin

^f^1 Aztreonam, 1 doxycycline, 1 eravacycline, 3 tigecycline, 1 polymyxin B, 2 metronidazole, 2 ciprofloxacin, 2 levofloxacin, 1 rifampin, 1 fluconazole

### Patient experience

Eighty patients provided information on their experiences with OPAT. Patients rated the overall effectiveness of ID provider communication of the plan prior to discharge, on a scale from 1 to 10 (10 being most effective), an average of 8.5 (*n* = 76). Most patients (78/80, 98%) indicated that they understood why they needed intravenous instead of oral antimicrobials. Only 2 of 80 patients (3%) stated that they had to visit the hospital owing to side effects from their intravenous antimicrobials. Eighteen of 80 patients (23%) reported receiving antimicrobials outside of the home; six of these 18 patients (33%) cited excessive cost as the reason. Overall, 55 of 80 patients (69%) reported spending ≤ 2 h per day administering or receiving intravenous antimicrobials at home, 18/80 (23%) spent 2–4 h per day, 2/80 (3%) spent > 4 h per day, and 5/80 (6%) did not respond. Overall, the OPAT process was well received, although some patients reported frustration with some aspects.

## Discussion

To our best knowledge, this is the largest study of OPAT outcomes in patients with solid tumors reported in the literature. We found that the types of infections treated for our patient population differed from previous reports, even from our own institution [2–6, 8–11, 14, 21–23, 31, 32]. Although we noted improvements in the completeness of ID provider recommendations and in laboratory test result monitoring during our project period, improvements in follow-up in the ID clinic and 30-day OPAT-related readmissions did not reach statistical significance.

Our initial intervention resulted in more complete recommendations by our ID providers regarding follow-up in the ID clinic and laboratory test result monitoring and in improved transitions of care. This intervention, however, did not include efforts to improve our ability to obtain laboratory test results. Because laboratory test result monitoring requires participation from multiple parties external to our ID clinic, our impact was limited without first engaging those stakeholders. Thus, our second quality improvement intervention aimed to achieve broader multidisciplinary engagement to improve acquisition of recommended laboratory test results. Through these combined efforts, we were able to improve laboratory test result monitoring and noted that follow-up in the ID clinic increased, albeit insignificantly. Although approximately 50% of patients still did not have complete laboratory test results available for ID provider review, we were able to reduce the number of patients who had no laboratory test result monitoring throughout the intervention periods. Although multidisciplinary coordination was necessary to improve laboratory test result monitoring, improvement in ID clinic follow-up was facilitated by internal improvements.

Recent studies regarding laboratory test result monitoring have shown that obtaining any, not necessarily weekly, laboratory test results led to a decreased risk for readmission [8]. Thus, our observed reduction in the number of patients with no laboratory test results may help reduce readmissions, and we observed trends supporting this hypothesis. That our observed decreased rate of readmissions did not reach statistical significance could reflect the low sample size of this pilot study. However, the degree to which readmissions are preventable among oncology patients has been called into question previously [33]. Other studies have shown that hospital readmissions among oncology patients may be modestly reduced by improving transitions from inpatient to outpatient care with similar interventions to those described here [34]. Those studies showed that even a 4.5% decrease in readmissions could significantly impact costs, suggesting that significant cost savings could result if our observed decrease in readmissions were confirmed by continued analysis. We further identified potential risk factors associated with readmission, including cancer type (lung and gynecologic), ECOG-PS, and type of vascular access. These relationships will need to be investigated further; however, these patients may warrant increased monitoring in an OPAT program or may necessitate prioritization when resources are limited.

Nearly one-third of our patients were treated with OPAT for abscesses, a larger percentage than has been previously reported in the literature, where common indications for OPAT include bacteremia and bone and joint infections [2–6, 8–11, 14, 21–23, 31, 32]. Our patient population often had medical comorbidities, which limited their ability to undergo source control procedures and possibly predisposed them to recurrent admissions for ongoing infection. Other notable differences regarding OPAT indications included a higher rate of genitourinary infections requiring OPAT compared with most previous reports. Our patients often had anatomical abnormalities with urinary devices present, likely predisposing them to recurrent and possibly multi-drug-resistant infections. Interestingly, fewer of our patients experienced MDRO or MRSA infections than reported by other groups [35]. The reasons for choosing OPAT in our patients should be explored further.

Regarding costs, although we noted a decrease in charges during the initial phase 1 intervention, it was not sustained during the post-phase 1 intervention or during the phase 3 intervention and after the phase 3 intervention. Given that this project occurred over a > 2-year time frame, charges may have increased over time as patients may have received novel, more expensive therapy. A potentially more informative outcome for this population may be time to return to intended oncologic therapy, because therapy may be delayed because of the infection [36]. Increased charges could represent a return to cancer therapy, signaling successful treatment of the infection.

Our study has some limitations. As a quality improvement project, there are limitations in the ability to generalize our findings to other treatment settings, and our findings are unlikely to be generalizable to non-cancer patients or even to patients with hematologic malignancies. However, this information may assist in developing OPAT programs for solid tumor patients at other cancer centers. Additionally, some bias may have resulted from the admission of some types of solid tumor patients to the oncology hospitalist service, resulting in exclusion from our analysis. By chance and because of the retrospective nature of the analysis, our phase I cohort does differ from our other cohorts. However, these differences are mitigated by our later cohorts and will likely continue to be mitigated over time and with the inclusion of additional patients. Further, during the project period, admission flow changed in our institution: patients with certain solid tumor diagnoses, not sarcoma, were admitted to the solid tumor service early in our project period but to our hospitalist service later. This change could have contributed to the increase in the percentage of patients with sarcoma during our project period. However, the impact of the admission flow change on the types of patients treated with OPAT was likely minimal, because overall indications for OPAT and other patient factors did not change significantly throughout the project period. Our rates of daptomycin use are higher than previous reports in the literature and likely reflect an institutional preference for daptomycin over vancomycin in the outpatient setting because of its daily administration, ease of monitoring, and toxicity profile rather than differences in antimicrobial sensitivity profiles.

Because 30-day outcomes and primary outcomes were evaluated through chart review, we were limited in our ability to capture readmissions and EC visits to outside institutions. Efforts were made to review all notes from patient encounters documenting outside hospital visits along with shared records within the EMR; however, some visits may not have been documented in this way. Thus, we may have underestimated our 30-day rates of EC visits and readmissions. Unfortunately, many patients were missing data on cancer stage and ECOG-PS, which may be associated with readmissions, which may have resulted in bias in estimating these risk factors. We will need to continue to monitor these trends in future cohorts to determine whether these factors remain predictive. Also, because patients frequently travel to visit our hospital, we were often unable to capture visits with local ID physicians, possibly underestimating rates of ID follow-up. Nonetheless, our population is motivated to follow up within our institution, so these instances are likely few and impact on our conclusions is likely minimal. Over half of the patients were discharged with home health agency assistance, and some patients reported having laboratory tests administered whose results were not noted in the EMR at the time of chart review. These limitations might also result in underestimation of laboratory test result monitoring in our patients, but these laboratory test results are useful only if they are available for review. Furthermore, past studies showing an association between laboratory test result monitoring and decreased readmissions accounted only for patients whose laboratory test results were available for physician review [8]. The incidence of patient adverse events may be underestimated as we relied on patient report through follow up phone calls or through review of the medical record.

Our patient survey data are limited because they were obtained from a convenience sample of patients who followed up in our ID clinic and because we have no baseline from prior to our OPAT interventions for comparison. Data collection on adverse events and antimicrobial classes was limited in that it was initiated during later intervention phases and could have changed over time. Regarding adverse events, over half of patients received both intravenous and oral therapy, making it difficult to discern whether adverse events were entirely attributable to their intravenous therapy. Chart review did not always reveal whether a central line was in place solely for OPAT or for an alternative indication. When this indication was unclear, central line–associated bloodstream infections were considered OPAT-related infections, which may have overestimated the number of central line–associated bloodstream infections directly attributed to OPAT.

## Conclusion

Infections among patients with solid tumors discharged with OPAT may differ from those reported among other OPAT patients. Through standardized recommendations, multidisciplinary engagement, and sustained efforts, monitoring of patients with solid tumors discharged with OPAT can be improved. As has been described in the general population, as laboratory test result monitoring improved, we observed somewhat fewer readmissions, suggesting that OPAT programs targeted toward patients with solid tumors are likely to improve the safety of OPAT.

## Data Availability

Upon request.

## Code availability

Upon request.
